# The public’s perceptions and attitudes of male nursing postgraduate professional identity on Chinese social media: Qualitative study based on machine learning

**DOI:** 10.1371/journal.pone.0331379

**Published:** 2025-09-04

**Authors:** Qi Zhou, Tao Zhou, Jing Huang, Yuling Lei, Guangxin Xu, Yuting Yang, Yanbing Shen, Yueli Zhu

**Affiliations:** 1 Integrated Traditional and Western Medicine Hospital of Linping District, Hangzhou, China; 2 The Fourth Affiliated Hospital of School of Medicine, Zhejiang University, Hangzhou, China; 3 Wenzhou Medical University, Wenzhou, China; 4 School of Nursing, Hangzhou Normal University, Hangzhou, Zhejiang Province, China; 5 School of Medicine, Zhejiang University, Hangzhou, China; Kwame Nkrumah University of Science and Technology, GHANA

## Abstract

**Background:**

Professional identity plays a critical role in the career development of male postgraduate nursing students, particularly in contexts where gender imbalance and social stereotypes persist.

**Objective:**

This study explores how the clinical professional identity of male nursing postgraduates is perceived and constructed through social media discourse in China.

**Design:**

A qualitative study using content analysis of social media discourse, supported by sentiment classification and clustering algorithms.

**Methods:**

Online comments related to male nursing postgraduates were extracted from Weibo and Zhihu. The data search was conducted from 2020 to 2023. This study was divided into five steps: data acquisition, data cleaning, statistical analysis, sentiment analysis, and topic analysis. Sentiment analysis was performed using a lexicon-enhanced rule-based model. Topic analysis was conducted using unsupervised machine learning.

**Results:**

Initially, 7,483 comments were collected. After cleaning, 5,692 valid comments totaling 486,366 words were retained for analysis. The sentiment distribution showed 64.3% were negative, 21.5% neutral, and 14.2% positive. Topic modeling revealed six main themes: identity confusion, gender role conflict, lack of clinical recognition, professional value affirmation, social support, and resistance to stereotypes.

**Conclusion:**

Public discourse reflects both affirmation and marginalization of male postgraduate nurses in China. These perceptions shape their clinical professional identity and influence their sense of belonging and future career planning. Interventions in education and media strategies are necessary to promote inclusive and supportive identity development.

## 1. Introduction

Nursing professional identity is a construct that nurses understand, internalize, and express in their roles [1]. Nursing has been a predominantly female profession, influenced by cultural and societal norms that shape both public perceptions and individual self-concept [2,3]. As healthcare environments evolve and social media plays an increasingly prominent role, professional identity is transforming, especially within underrepresented groups such as male nurses [3]. In this study, ‘postgraduate nursing students’ refer specifically to those who have completed undergraduate nursing education and are currently pursuing a master’s degree in nursing. Although these individuals possess formal clinical training, they often encounter ambiguity regarding their professional role and identity. This situation is largely due to the absence of differentiated clinical pathways for postgraduates in China, combined with persistent gender-role expectations in a female-dominated field. As such, male postgraduate nurses may simultaneously be perceived as insiders by virtue of their clinical training and education, yet also as outsiders within the gendered norms and structural limitations of the profession. This dual status—being professionally credentialed but socially marginalized—produces a unique form of identity tension that challenges their self-concept and affects their engagement in clinical roles and future career planning [2].

In the global shortage of nurses, the shortage of male nurses is more pronounced than that of female nurses [4]. Among developed countries, the proportion of registered male nurses is 11.1% in Australia, 10.7% in the UK, and 9.1% in the US [5]. However, in China, despite having a large nursing workforce, male nurses make up only about 2% of the total number of registered nurses, with high attrition rates [6]. This situation raises critical questions: Why is there such a high attrition rate among male nurses in China? Is it related to societal attitudes toward male nurses, which deeply ingrained gender stereotypes may influence Chinese society? These societal biases often lead to male nurses facing additional pressures, including limited role models, negative public perceptions, and challenges in balancing professional and personal identity [7].

Maintaining a high-level professional identity in nursing means that a nurse has a clear, committed, and positive sense of self within their professional role, recognizing their value and contribution to the healthcare system [8]. It encompasses a strong sense of belonging to the nursing profession, pride in one’s work, and the ability to navigate complex clinical situations with professionalism and confidence [1]. A high-level professional identity is associated with higher job satisfaction, work engagement, and career longevity. Nurses who maintain a strong professional identity are more likely to provide high-quality patient care and contribute to the development of the nursing field [9]. In contrast, a weak professional identity can lead to role ambiguity, disengagement, and burnout. This problem is especially concerning in postgraduate nursing education, where the focus on academic and clinical skills often overlooks the development of professional identity [9]. In China, the postgraduate nursing education system is still in its early stages and often lacks structured content related to professional identity development, particularly with regard to gender diversity in nursing. This gap means that postgraduate students, particularly male nurses, may struggle to reconcile their personal and professional identities, which can contribute to higher attrition rates and lower career satisfaction [10].

In developed countries, postgraduate nursing programs incorporate professional identity development into their curricula as an essential component of nurse education. In the United States, nursing programs emphasize the importance of leadership, role modeling, and reflective practices to foster professional identity among postgraduate students [3]. Similarly, in the United Kingdom, nursing education integrates professional identity development by providing postgraduate students with the tools to reflect on their roles as practitioners and leaders [11]. By contrast, in China, the emphasis in postgraduate nursing education is predominantly on clinical and technical competencies, with little attention paid to professional identity development. This gap is particularly evident in male nursing students, who often face additional societal pressures and gender stereotypes that are not adequately addressed in their academic training [12]. As a result, many male nurses may feel disconnected from the profession, which can lead to dissatisfaction and high turnover rates [9].

The gender factor should be as crucial as social status, salary, and academic level in the professional identity education of postgraduate nursing students [12]. Researchers in developed countries have begun to focus on the professional identity of male postgraduate nursing students [13]. Many studies have shown that this group has a solid professional identity [14,15], and only a few researchers have mentioned that they have problems with reality-expectation incompatibility and Negative attitudes towards nursing among other medical students [16]. However, several studies have found that the clinical professional identity of Chinese postgraduate nursing students is low under the Chinese education system [17,18]. Some scholars contend that many Master of Professional Nursing students are unwilling to choose clinical nursing as a career choice due to their perceived inability to prove their worth [19,20]. A low sense of professional identity causes postgraduates to be confused about their postgraduate life, lack a commitment to their role, make ineffective use of educational resources, fail to achieve disciplinary education goals, and finally, impact disciplinary construction and development [10,19–21].

Female postgraduate students dominate professional identity studies and clinical career development planning in China and need to adequately reflect the career development needs of male clinical nursing postgraduate students [20]. Therefore, we must concentrate on the professional identity of Chinese male nursing postgraduates, determine the issues and corresponding responses to their Choice of clinical nursing work, highlight their significance, and encourage more men to choose clinical careers. Similarly, this study can inform nursing educators in underdeveloped countries and different social models.

According to Mao’s analysis of the Chinese national context, the professional identity of nurses and nursing students is influenced by the micro, medium, and macro dimensions [7]. Personal factors at the micro dimension and family and institution factors at the macro dimension can all positively and negatively influence professional identity. However, social factors in the macro dimension tend to harm professional identity [7]. The negative results concluded by the Chinese researcher solely through qualitative interviews with patients and other medical professionals need to represent the public’s views adequately [19,20,22,23]. Guraya and ten Hoeve also pointed out that in the digital era, negative social media comments will harm healthcare professionals’ professional identity [24,25]. However, Chinese researchers ignored this factor [26]. In conclusion, there is a research gap in Chinese social media regarding whether the public perceives the clinical career choice of male nursing postgraduate students positively or negatively.

In the digital age, social media has become a powerful tool influencing professional identity development. Platforms like Weibo and TikTok are shaping public perceptions of various professions, including nursing [27]. Negative portrayals of male nurses or gender stereotypes perpetuated through social media can significantly impact the professional identity of male nurses, especially in countries like China, where societal expectations are heavily influenced by digital content [3]. Despite the growing role of social media in shaping professional identity, Chinese research on its impact remains sparse. Research from other countries has shown that social media can either reinforce or challenge gender stereotypes, making it a critical factor in shaping the career paths and professional identity of male nurses [27]. Therefore, based on data from the Chinese social media platform and using sentiment and clustering algorithms, this study will analyze the attitudes and perceptions of the public regarding the clinical career identities of male nursing postgraduate students on the Internet and provide suggestions for improving clinical professional identities.

This study aims to address this gap by exploring how social media influences the professional identity of male nursing postgraduates in China. By analyzing sentiment and discourse on social media platforms, this research will examine how the public perceives male nurses and how these perceptions impact their professional identity development. The findings will provide valuable insights for nursing educators and policymakers, highlighting the need for more inclusive educational practices and social media strategies that promote a positive professional identity for male nurses.

## 2. Methods

This study adopted a qualitative design that integrates computational context analysis and machine learning techniques to examine public perceptions of male postgraduate nursing students. This integration method is increasingly adopted in health informatics research to derive meaningful insights from public discourse.

### 2.1. Data acquisition

We chose the Weibo and Zhihu platforms as the data sources for this study [28]. Weibo and Zhihu are two major Chinese social media platforms. Weibo functions similarly to Twitter (now X), allowing users to post short messages and engage through reposts and comments. Zhihu is comparable to Quora, structured around question-and-answer threads that often generate in-depth discussions. These platforms were selected due to their wide reach, active user engagement, and openness to public search, making them ideal for analyzing public sentiment. The data search was conducted from 2020 to 2023 using keyword combinations such as “male nurses,” “male postgraduate nursing students,” and “nursing.” Searches were performed manually. The search process took approximately three weeks and was conducted concurrently on both platforms. No personal identifying information was collected. Data was obtained from publicly accessible domains and was consistent with ethical guidelines for internet research. All retrieved data were anonymized and stored. Data management followed best practices for reproducibility and integrity, including structured file naming and version tracking.

### 2.2. Data cleaning

Python is a programming language widely used in data science. Libraries such as RE (for regular expressions), Jieba (for Chinese text segmentation), NLTK (for stop word removal), Pandas (for data manipulation), and BeautifulSoup (for HTML parsing) were employed for preprocessing. Python enables automation and standardization of cleaning steps across large datasets. This function is particularly useful when dealing with noisy social media data. Two researchers manually read all the texts and picked out comments that were not relevant to the topic, such as advertisements. When disputes were encountered, a third researcher was requested to make a judgment and reach a consensus. The Python function was divided into seven steps.1) Used the RE library to remove special characters and punctuation.2) Used the Jieba library for Chinese text segmentation to ensure the text is correctly split into words.3) Used the NLTK library to remove the stop-words.4) Used RE and DateTime libraries for redundant numbers and dates.5) Used the lower function to de-unify the text case. 6) Used the panda’s library to remove blank text and fill with NULL for this text, mainly the personal introductions. 7) Used the BeautifulSoup library to remove HTML labels.

### 2.3. Statistical analysis

Statistical Analysis consisted of time and personal analysis. Time analysis was used to verify public perceptions and attitudes toward the professional identity of male nursing graduate students, changes over time. We used the DateTime and collection libraries to analyze when comments were posted or edited. Personal Analysis was used to identify the source of views, distinguishing between the public, other medical staff, and the male postgraduate nursing students. We used the collection library for the initial screening and manually verified this result for accuracy.

### 2.4. Sentiment analysis

Sentiment analysis validated user judgments about attitudes toward clinical careers and yielded experimental text on positive and negative sentiments. We cross-validated results using both models, performed a manual review on a random sample of comments, and ensured dataset balancing by selecting comparable volumes of positive and negative instances during model training. Comments have many positive and negative attitudes, so we consider balancing Precision and Recall to get a better F1 score [29]. Ultimately, the F1 scores of the two algorithms are more than 75, which satisfies the requirements of the algorithms.

The Bayesian algorithm was divided into three different steps in this study [30,31]. First, we perform feature extraction to transform the textual data into feature vectors using word embedding methods. This step converts the textual information into numerical features for processing by the Bayesian algorithm. Second, we choose the plain Bayesian classifier, whose features can be independent based on Bayes’ theorem. This step simplifies the model and makes it computationally efficient. Third, we use the labeled training data to train the Bayesian classifier to obtain the probability distribution of the sentiment categories. Finally, when the model reaches a satisfactory performance, we use the model to predict the experimental text for sentiment analysis.

The Boson NLP was divided into three steps: matching the sentiment lexicon, calculating the sentiment score, and categorizing the sentiment [28]. First, the Matching Boson NLP Sentiment Dictionary comprises negation, degree adverbs, and deactivation dictionaries. Second, the Boson dictionary calculates text sentiment scores by multiplying sentiment words by their number. Finally, sentiment was categorized with scores less than 0.50, indicating negative attitudes, and scores more than 0.50, indicating positive attitudes.

The Bayesian and Boson NLP methods were selected due to their complementary strengths—Bayesian classifiers are data-driven and adaptable, while Boson NLP provides a lexicon-based sentiment framework tailored for Chinese text. Other potential methods, such as SVM, LSTM, and BERT, were considered but were not chosen due to the limited labeled data and higher computational cost, which did not align with our project scope.

### 2.5. Topic analysis

Topic algorithms validated public concerns regarding postgraduate men’s nursing students’ clinical careers. Study data was sourced from the Internet, which is large and complex. In addition, the Latent Dirichlet Allocation topic clustering algorithm (LDA algorithm) is highly flexible, allowing the number of topics to be adjusted according to the task [32]. Therefore, we use the LDA algorithm to discover potential topics from the experimental text. The LDA algorithm has been validated as effective for engineering, medicine, geography, and political science [32–35]. The steps of the LDA algorithm in this study were divided into five steps [34,35]. First, the text data is converted into a document-word matrix, where each row represents a document, each column represents a word, and the matrix elements represent the frequency or weight of the word in the document. Second, set the hyperparameters of the LDA algorithm, including the number of topics and iterations. Third, train the LDA algorithm: The LDA algorithm is based on a probabilistic graphical model. Each document is generated from several topic word distributions, and each topic word distribution is generated from several clustered words. Fourth, the optimal number of topics in LDA modeling was determined using coherence scores and perplexity metrics. We evaluated topic coherence across models with 5–20 topics and selected the model with the highest coherence and interpretable clusters. After completing the training, we can get the distribution of different topics and clustered word distributions. Finally, we used content analysis to interpret the topic distributions to increase the understanding and credibility. We manually reviewed the representative keywords and top-ranked comments within each topic to inductively generate descriptive labels and interpret meanings.

### 2.6. Ethics approval

Although no direct interaction with human subjects occurred, this study adhered to institutional and national ethical standards for online data use. We submitted the protocol to the Institutional Review Board of Integrated Traditional and Western Medicine Hospital of Linping District, which confirmed the exemption status. All data collected was from a public platform. This study includes 5,692 comments (3,207 comments came from Zhihu and 2,485 from Weibo), and the collection and analysis method complied with the terms and conditions for the source of the data. Data use complied with the platforms’ user agreements and relevant ethical guidelines for internet-based research. This study needs the requirements of the checklist *Guidance for publishing qualitative research in informatics* [36].

## 3. Results

### 3.1. The result of data cleaning

Initially, 7,483 comments were collected. After cleaning, 5,692 valid comments were retained for analysis, totaling 486,366 words. The 3,207 comments came from Zhihu and 2,485 from Weibo.

### 3.2. The result of statistical analysis

Based on time analysis, we found that these questions and responses were clustered in the last three years, with 2593 in 2020, 1096 in 2021, and 844 in 2022. Based on personal Analysis, we found that the primary participants were the public, and supplemented participants were healthcare professionals, with 115 comments from male postgraduate nursing students.

### 3.3. The result of sentiment analysis

The results of the two algorithms are similar. The Bayesian algorithm yielded 66.3% positive, 28.8% negative, and 4.9% objective texts. The Boson algorithm yielded 65%% positive texts and 35% negative texts. The results of the experimental texts’ sentiment analysis were categorized into 3687 positive comments and 2005 negative comments.

The positive and negative texts are as follows.

Positive texts

1: *Skilled, pragmatic, and progressive, the future is promising; go for it!*
2: *It is very popular. It is easier to find a job than a girl.*

Negative texts

1: *It is inappropriate and annoying with women. You could be a teacher instead of a clinical nurse.*
2: *Nursing could be a better major. I urge you to change your major.*

### 3.4. The result of topic analysis

Based on LDA topic modeling and manual interpretation, six main themes emerged from the discourse: (1) identity confusion, with users questioning the legitimacy or clarity of male nurse roles; (2) gender role conflict, highlighting social biases; (3) lack of clinical recognition, especially in high-intensity settings; (4) professional value affirmation, reflecting public support and appreciation; (5) social support, demonstrating peer or family encouragement; (6) resistance to stereotypes, user defends career choice of male nurse (Table 1).

**Table 1:** The result of the topic analysis.

Source	Subject Terms	Topic Share
Agree	management post, emotional intelligence, valued, regimental favorites, establishment, promotion, research capacity, special, performance opportunity, stable, favorite	51.3%
Oppose	discrimination, jealousy, Discriminatory vision, salary, marriage, earning, expensive, college, tertiary, Ph.D., colleagues, heavy workload, exhausted	48.2%

Through clustering and sentiment analysis, this study determined that most respondents had positive attitudes toward the career growth of male nursing postgraduates in clinical settings, particularly in career choice and job description. Those who responded negatively believed that highly educated male nurses should have more opportunities and not be limited to clinical practice, particularly in employment opportunities, work experience, career choices, and career advancement (Table 2).

**Table 2:** The result of the content analysis.

Attitude	Topic	Explanation
Positive	Career options	Male nurses with a postgraduate degree are a rare high-level talent in hospitals and will be favored by hospital managers.
	Job Description	Male nursing postgraduates will have greater opportunities for clinical nursing research, to present their nursing research, to attend academic lectures, to engage in training programs, etc., as well as more chances to receive training abroad.
Negative	Employment Opportunities	A bachelor’s degree is adequate to fulfill the academic criteria of tertiary hospitals. Clinical roles for postgraduate students in hospitals would be a waste of qualifications. In addition, the hospital administration lacks a defined training program.
	Work experience	The inability to utilize their academic advantages in clinical nursing. Excessive workload, irregular working hours, night shift issues, medical nursing interactions, nurse-patient relationships, and departmental issues prohibit male nursing postgraduates from obtaining clinical roles.
	Career choices	Some male nursing students believe the clinical practice to be stressful and their social status to be low in terms of career prospects.
	Career Advancement	Lack of real-life examples and misunderstanding of the promotion route in clinical work contribute to a lack of confidence in clinical promotions.

#### 3.4.1. Positive views.

The public believed that the male nursing postgraduates’ advantages in clinical professional identities were primarily related to career choices and job satisfaction.

Career choice: The public believes that male nurses with postgraduate degrees are in short supply and will be treated favorably by hospital administrators. However, female nursing postgraduate students mentioned that male nursing postgraduate students are taking away their employment opportunities.

Netizen A: *Most significant hospitals demand a bachelor’s degree. Hospital positions favor postgraduate degrees over bachelor’s degrees*.

Netizen B: *Demand for nurses is rising as most tertiary schools require a bachelor’s degree. Postgraduate nursing students have more contract options than undergraduate nursing students*.

Job description: Male nursing postgraduates have more opportunities to conduct research in clinical nursing work than their female counterparts. They have more significant opportunities to train worldwide and can participate in nursing research presentations, academic lectures, and training programs.

Netizen C: *Nurses with more education have more chances to work on hospital research, patent applications, and editing books, which means we can grow*.

Netizen D: *If a nursing postgraduate student is talented or hardworking in their research or project and achieves good results, the hospital or department will award research money, and many peers will seek help in writing title assessment papers (they are all paid). At least your earnings will be high, and your title will rise fast*.

#### 3.4.2. Negative views.

The public believed that the male nursing postgraduates’ concerns in clinical professional identities were primarily related to employment opportunities, work experience, career advancement, and career choices.

Employment Opportunities: Regarding employment opportunities, the public believes a postgraduate degree is optional because a bachelor’s degree satisfies the educational requirements of major tertiary hospitals. However, male postgraduate students found it challenging to find jobs. Due to the hospital administration’s lack of a standardized training program, male nurses with postgraduate degrees will not be qualified for hospital clinical positions. This situation will necessitate increased professional fulfillment.

Netizen E: *Not good! Do not come and study! It is no use at all! It is a waste of youth. Why not go straight to work in a hospital and make money? You can find an undergraduate male nurse job, so why study? You will be able to learn to write articles in the department; articles on a few years of classes to gradually follow can also feel out some of the way.*

Netizen F: *You can find many people who have been in the business for a long time.*

Work experience: Regarding work experience, most male nursing postgraduates considered their clinical work environment equivalent to nurses in their undergraduate specialties. Their lack of clinical practice skills and practical confidence compared to nurses of the same age explains why they must apply their academic skills in clinical nursing. In addition, challenges such as too much work, unusual hours, night shifts, doctor-nurse relationships, nurse-patient relationships, and departmental issues make it difficult for male nursing postgraduates to switch to clinical employment.

Netizen G: *Graduate school is the most worthless and requires two years of clinical rotation. Imagine doing the night shift with youngsters at 30.*

Netizen H: *Oral care, perineal care, lower urinary catheter, enema, these are not yet you tired?*

Career choices: Some male nursing students consider the clinical practice to be time-consuming and low-status. They studied in order to escape the clinic and achieve social status. Fear of clinical pressure, recommendations from parents and relatives, and a desire for a higher social status led these male nursing students to choose a non-clinical role, such as clinical research associate (CRA), clinical research coordinator (CRC), university instructor, civil servant, etc.

Netizen I: *You can switch to a medical-related career or become a nursing teacher. You can also work in clinical research, such as CRA or something like that.*

Netizen J: *Are you still planning to go into clinical? No, you can be a teacher!*

Career advancement: Male postgraduate nursing students required additional information regarding career advancement. Clinical development is viewed with skepticism due to a need for actual examples and comprehension of its routes. In addition, as male nurses age, their family responsibilities rise, and they must determine if their pay is sufficient to support their families.

Netizen K: *Do not be stupid and assume a postgraduate student can get into nursing; if you do, you are a handyman, and the hospital cares about seniority and business.*

## 4. Discussion

This study aimed to explore how male nursing postgraduates’ clinical professional identities are perceived and constructed through social media discourse. The key findings revealed both perceived professional advantages and structural challenges, categorized into six major themes: identity confusion, gender role conflict, lack of clinical recognition, professional value affirmation, social support, and resistance to stereotypes.

### 4.1. Gender hinders a solid clinical professional identity

The positive results of career choices and job descriptions indicate that the public on social media believes male nursing postgraduate students have an advantage in choosing a clinical career. However, the negative result of employment opportunities indicates that the nature of these advantages is due to gender and is the same as the difference between male and female nurses in general and specialist nursing. Male nursing postgraduates tend to be assigned to high-intensity departments. Such departments require not only male nurses’ height and physical strength but also the ability to respond to unexpected situations and adapt in the face of high-intensity work [37]. This distribution pattern is not unique to postgraduates; male nurses with only bachelor’s degrees also frequently enter these departments, creating competition across educational levels. Therefore, gender-based task assignments result in structural employment pressure for all male nurses, regardless of their academic credentials. However, as the number of male nursing postgraduates increases, the employment advantage of male postgraduates due to their small number and gender will gradually decrease, eventually leading to recruitment hospitals raising the entry barrier to make employment more difficult. Therefore, male nursing postgraduates—despite already having prior nursing education—should reflect critically on their career positioning within a system where academic qualifications do not yet correspond to differentiated clinical roles [38].

While male postgraduate nursing students demonstrate visible confidence and clinical presence on social media, this perceived strength does not necessarily translate into a sustainable professional identity due to gendered role assignments and increasing competition. From a social identity perspective, male nursing students may experience a sense of role conflict due to their minority status within the profession, which complicates their identification with the nursing group. Gender stereotypes in nursing are prevalent in China and many Western countries. However, in many Western countries, male nurses are seen as more integrated into the profession, with increasing gender diversity in nursing leadership roles [11]. This contrast suggests that cultural attitudes toward gender roles significantly influence the professional identity and career trajectories of male nurses. Additionally, male nurses already working in the field may feel marginalized or excluded, which can affect their job satisfaction and ultimately lead to higher turnover rates [9,12]. Healthcare institutions need to address these perceptions through targeted recruitment strategies and inclusive workplace policies. In China, the development of gender-inclusive policies and public awareness campaigns is critical to overcoming these barriers and improving the recruitment and retention of male nurses [7,12]. Public awareness campaigns should be launched to challenge and reduce stereotypes surrounding male nurses. By promoting positive role models and highlighting the contributions of male nurses, these campaigns can help reshape societal perceptions.

In sum, the positive portrayal of male postgraduate nursing students on social media can serve as a double-edged sword. It can be leveraged as a tool for public health messaging and nursing recruitment, showcasing the capability and professionalism of male nurses. On the other hand, if not aligned with institutional policies and clinical realities, it may result in unmet expectations or role confusion among male students. Health institutions and policymakers should consider utilizing these social narratives to improve recruitment strategies that highlight male role models in clinical leadership or specialized care settings.

### 4.2. Career advancement mechanisms reduce the professional identity

The negative results of career choices and career advancement implied that career advancement mechanisms hinder male postgraduate nursing students from choosing clinical jobs. Currently, nurses in China have a rather exclusive path to career advancement. According to the category classification, nursing management positions are categorized from lowest to highest as follows: chief nurse, department head nurse, nursing department officer, deputy director of the nursing department, and director of the nursing department, whereas according to the title level classification, these positions are categorized as follows: nurse, nurse practitioner, supervisor nurse practitioner, and deputy director nurse practitioner/director nurse practitioner [39]. The answer screening results of this study revealed that fewer male postgraduate nursing students earned senior titles or management positions, resulting in a low level of professional identity for male nursing research. This occurrence may be attributable to the poor development of the professionalization of male nurses in China and the fact that most male nurses still hold entry-level to mid-level positions after a brief period of employment [40].

Meanwhile, postgraduate male nursing students are more eager to obtain financial rewards and faster promotion due to their lack of income and ability to advance during their education. According to Holland’s theory, individuals seek careers that match their interests and values. The lack of clear advancement pathways for male postgraduate nurses may create a mismatch, reducing their motivation to remain in clinical roles. This situation has led some male postgraduate nursing students to have a lower professional identity and choose positions with clear career progression, such as teaching and civil service. The current advancement systems do not accommodate the developmental needs of male postgraduate nurses, which directly weakens their professional identity and may redirect them to non-clinical paths. Developmental needs of male postgraduate nurses include fair access to title promotion, scientific research platforms, and involvement in management pathways. In contrast, postgraduate nurses in countries like the UK or the US benefit from mentorship, dual academic–clinical appointments, and flexible promotion routes, which collectively strengthen their identity and retention in clinical practice [11]. Therefore, the author recommends that managers scientifically and effectively support the reform of title promotion processes to better stimulate the job enthusiasm of male nursing graduate students [41]. Secondly, administrators organize more activities, such as the Male Nurses Union and the Male Nurses Forum, arrange for male nursing postgraduates to communicate with their nursing seniors and increase the opportunities for male nursing postgraduates to participate in the daily management of nursing administration and the development of specialties within a reasonable frame [40–42]. Finally, establishing mentorship programs for male nurses can play a significant role in supporting career advancement. These programs can help male nurses navigate career challenges, develop professional skills, and gain confidence in leadership roles.

### 4.3. The contradiction between the realistic nature of the work and development expectations reduces the professional identity

The negative results of career advancement and work experience reveal that male postgraduate nursing students had high career expectations but low job satisfaction.

This disconnection between training and reality creates not only disappointment but also emotional disengagement from the clinical profession. When male postgraduate nurses perceive that their education cannot be applied meaningfully, their sense of accomplishment is diminished, and they are more likely to consider leaving clinical roles. Before joining clinical nursing, they believed they had the foundation to become specialist nurses and could take on more excellent administrative and research responsibilities. However, in reality, the clinical work of male nursing postgraduates is still mainly essential nursing work, and management and research work are only additional supplementary work for them. The authors think that the issue of stratification should be tackled primarily at the national policy level. While hospitals can innovate in job design, the lack of standardized national guidelines severely limits institutional autonomy in advancing clinical roles. This view is similar to the findings of Cai Chan and Li Peng et al. [43,44]. Cai Chan pointed out that the late start of postgraduate education in China has resulted in ambiguous clinical positioning [43]. Peng et al. observed that without systematic integration of research and clinical responsibilities, postgraduate nurses struggle to align career expectations with real-world practice, leading to lower identity coherence [44]. The late start of postgraduate nursing education in China compared to foreign education led to more explicit clinical orientation and future career paths. Secondly, at the hospital level, male nursing postgraduates are essentially nurses. However, many male nursing postgraduate students focus on academic study while neglecting clinical skills and thus need more clinical experience. Hospitals need to train them from the clinical frontline again, like undergraduate specialist students, so that they can exercise their clinical practice level in clinical work. This phenomenon is consistent with the reasons for the problems of female postgraduate students investigated by Lu Hui [45]. Lastly, most hospitals in China need to set up professional research positions, and the scientific research and management knowledge learned during postgraduate studies cannot be well applied to clinical nursing work, which makes male nursing postgraduates feel low professional value and professional achievement. Therefore, the author believes the national level should improve nursing career stratification policies and management standards. Personalized training for male nursing postgraduates is also necessary for the professional subdivision of the nursing field. At the hospital level, hospital administrators could create roles for clinical research nurses to help them hone their management and research skills by following the example of Western nations like the UK, the USA, and Japan [46]. Therefore, the gap between educational attainment and practical role assignment undermines professional achievement and identity among male postgraduate nurses.

### 4.4. Limitations

This study is the first to use a machine learning approach to explore social media for male nursing postgraduate students. However, there are five limitations. First, all applied sentiment analysis algorithms may not be able to recognize nuances between contexts, so we choose two algorithmic and manual approaches to compensate for this shortcoming. Second, Internet users are young and middle-aged, lacking representatives of the older population. Third, some people in the experimental text needed a personal introduction, so this study could only analyze users who had completed a personal introduction. Fourth, we did not separately analyze the effect of COVID-19 on the male nursing postgraduates’ professional identity. Fifth, the amplification effect of social media means that negative incidents, such as stories of discrimination or exclusion, are often highlighted far more than positive experiences or institutional support. This selective reporting can lead to skewed perceptions of male nurses’ professional realities, which in turn affect their career choices and professional identity formation. Future studies should consider and address this actual limitation.

## 5. Conclusion

This study argues that the government should improve career stratification policies and management standards to address the disadvantages of employment opportunities, work experience, career advancement, and career choices. Hospitals and nursing administrators should create gender-friendly work environments, establish male nursing professional groups, provide individualized training programs, and reform promotion processes. Male postgraduate nursing students should improve their academic skills and clinical practice to demonstrate their professional competence.

**What is already known**

China’s inadequate postgraduate education system and neglect of professional identity education have confused nursing postgraduate students about their future in nursing.

In the digital era, negative social media comments will harm healthcare professionals’ professional identity.

**What this paper adds**

The public believed that the male nursing postgraduates’ advantages in clinical professional identities were primarily related to career choices and job satisfaction.

The public believed that the male nursing postgraduates’ disadvantages were employment opportunities, work experience, career advancement, and career choices.
